# Cognitive remediation and professional insertion of people with schizophrenia: RemedRehab, a randomized controlled trial

**DOI:** 10.1192/j.eurpsy.2021.25

**Published:** 2021-04-15

**Authors:** S. Cervello, J. Dubreucq, M. Trichanh, A. Dubrulle, I. Amado, M. C. Bralet, M. Chirio-Espitalier, S. Delille, E. Fakra, C. Francq, N. Guillard-Bouhet, J. Graux, C. Lançon, J. M. Zakoian, E. Gauthier, C. Demily, N. Franck

**Affiliations:** 1 Psychosocial Rehabilitation Resource Center (CRR) and Reference Center (SUR-CL3R), Le Vinatier Hospital, Lyon, France; 2 Psychosocial Rehabilitation Reference Centre, Alpes Isère Hospital, Grenoble, France; 3 Service hospitalo-universitaire, CJAAD, centre hospitalier Sainte-Anne, 75014 Paris, France; 4 Faculté de médecine, université Paris Descartes, Sorbonne Paris Cité, 75006 Paris, France; 5 Inserm, laboratoire de physiopathologie des maladies psychiatriques, centre de psychiatrie et neurosciences, U894, institut de psychiatrie (GDR3557), 75014 Paris, France; 6 Service hospitalo-universitaire, C3RP, centre hospitalier Sainte-Anne, 75014 Paris, France; 7 Crisalid Unit (FJ5), CHI Clermont de l’Oise, 2 rue des Finets, 60607 Clermont, France; 8 Pôle de psychiatrie et santé mentale, Centre de référence en soins d’éducation thérapeutique et remédiation cognitive (CReSERC), centre hospitalier universitaire, 85, rue Saint-Jacques, 44093 Nantes cedex, France; 9 Département de Réhabilitation Psychosociale et de remédiation cognitive, Lille, France; 10 University Department of Psychiatry, Centre Hospitalier Universitaire de Saint-Étienne, Saint-Étienne, France; 11 INSERM U1028, CNRS UMR5292, Lyon Neuroscience Research Center (CRNL), PSYR2 Team, Université de Lyon, Lyon, France; 12 Center of Research in Economics and Statistics (CREST), UMR 9194, Palaiseau, France; 13 CREATIV & URC Pierre Deniker, Centre Hospitalier Laborit, Poitiers, France; 14 Se rétablir 37, CHRU de Tours, UMR 1253, iBrain, Université de Tours, Inserm, Tours, France; 15 CEReSS, Université de la mediteranée, Marseille, France; 16 GénoPsy, Reference Center for Diagnosis and Management of Genetic Psychiatric Disorders, Centre Hospitalier le Vinatier and EDR-Psy Q19 Team (Centre National de la Recherche Scientifique & Lyon 1 Claude Bernard University), 69678 Bron, France; 17 UMR 5229, CNRS & Université Claude Bernard Lyon 1, Université de Lyon, Lyon, France

**Keywords:** cognitive remediation, professional insertion, RECOS, schizophrenia, vocational rehabilitation

## Abstract

**Background:**

People suffering from schizophrenia cannot easily access employment in European countries. Different types of vocational programs coexist in France: supported employment, sheltered employment (ShE), and hybrid vocational programs. It is now acknowledged that the frequent cognitive impairments constitute a major obstacle to employment for people with schizophrenia. However, cognitive remediation (CR) is an evidence-based nonpharmacological treatment for these neurocognitive deficits.

**Methods:**

RemedRehab was a multicentric randomized comparative open trial in parallel groups conducted in eight centers in France between 2013 and 2018. Participants were recruited into ShE firms before their insertion in employment (preparation phase). They were randomly assigned to cognitive training Cognitive Remediation for Schizophrenia (RECOS) or Treatment As Usual (TAU). The aim of the study was to compare with the benefits of the RECOS program on access to employment and work attendance for people with schizophrenia, measured by the ratio: number of hours worked on number of hours stipulated in the contract.

**Results:**

Seventy-nine patients were included in the study between October 2018 and September 2019. Fifty-three patients completed the study. Hours worked / planned hours equal to 1 or greater than 1 were significantly higher in the RECOS group than in the TAU group.

**Conclusions:**

Participants benefited from a RECOS individualized CR program allows a better rate of work attendance in ShE, compared to the ones benefited from TAU. Traditional vocational rehabilitation enhanced with individualized CR in a population of patients with schizophrenia is efficient on work attendance during the first months of work integration.

## Introduction

People with schizophrenia frequently express the need to find a job or to be helped to keep a job [1]. Despite individual and collective beneficial effects [2–5], there are very few people with severe mental illness in general and schizophrenia especially in employment [6,7]. There are wide variations in reported employment rates in schizophrenia. Most recent European studies report employment rates for people with schizophrenia between 10 and 20% [7–10]. People with severe mental illness are six to seven times more likely to be unemployed than people with no mental health problems [8].

For people with schizophrenia, obtaining and retaining employment translates into better social and family relationships, overall health, and self-esteem [11,12], resulting in quality-of-life improvements. Employment supports the recovery process, which needs empowerment, a restored self-esteem, a positive social identity, and an improved quality of life [3–5,13–16].

Despite these well-documented benefits, employment rate of people with schizophrenia remains quite low in France, and job opportunities are frequently located in sheltered employment (ShE) [17]. ShE or workshop approach is called the traditional or “train and place” vocational rehabilitation [2,17,18] and has longer been the only alternative to unemployment for people with schizophrenia [19]. It offers to people a training during a long period of prevocational training, with a small proportion obtaining competitive employment. Another approach is the “place and train” approach, such as supported employment (SE) programs, in which employment specialists or “job coaches” help people obtain and then keep a competitive job as fast as possible with no requested training [18,20]. The most effective vocational services are based on the “place and train” model, and even more precisely on the individual placement and support model, that allows to more than 50% of people with a severe mental illness to obtain a competitive employment after 6–18 months of individualized support [17,21,22].

There are different outcomes that can constitute obstacles to employment in schizophrenia. Social, demographic, environmental, or even personal factors can partially explain the poor access and maintaining in employment of people with severe mental illness. Factors relating to the disease itself are mostly involved. Negative symptoms have a substantial adverse impact in both competitive employment and ShE [23]. But it is widely acknowledged that cognitive impairments associated with schizophrenia are outcomes which most affect work access and maintaining [3].

Various domains of neurocognition assessed by individual tests are related to work functioning [24]. Level of cognitive functioning is a predictor of patients’ work outcomes [25]. Cognitive deficits in schizophrenia are unspecific and include deficits in attention, learning and memory, declarative and working memory, speed of processing, and executive functioning such as reasoning and problem-solving [26,27]. Ninety percent of people with schizophrenia have clinically meaningful deficits in at least one cognitive domain, and 75% have deficits in at least two [28]. Learning of new work tasks is affected by attention and memory impairments [27]. Executive dysfunction can lower the patient’s work functioning, resulting in a poorer work integration [27]. Executive functioning, verbal learning, attention, and psychomotor speed, as well as the severity of psychotic symptoms, are correlated with the needs of vocational rehabilitation [25]. Vocational rehabilitation appears to work by compensating for the effects of cognitive impairment and symptoms on work, according to a review of literature conducted by McGurk and Mueser [6].

Cognitive remediation (CR) is an evidence-based nonpharmacological treatment for the neurocognitive deficits frequently associated with severe mental illness [29]. CR is most likely to impact functional outcomes when individuals are given opportunities to practice their cognitive skills and is more effective when integrated in psychosocial rehabilitation programs and connected to patient’s goals [26,29–33]. In routine psychiatric practice, performing a neuropsychological assessment should be used systematically to clarify the project of care and rehabilitation of patients with schizophrenia. In addition, remediation should also be widely used therapeutically, and many psychiatric services have adopted it during the last years. CR associated with SE has demonstrated its cost-effectiveness compared to traditional vocational rehabilitation [34]. CR combined with SE or traditional vocational interventions appears to be more effective on work outcomes, such as work attendance, than SE only or other vocational interventions without CR [3,35–40]. It is particularly true for people with lower community functioning, as SE enhanced with CR improves their vocational outcomes, but this difference is not significant in higher-functioning participants’ groups [41]. Improvements in executive functioning and verbal memory could particularly predict improvements in daily functioning in schizophrenia [42].

Taking this into account, it is a major challenge for psychosocial rehabilitation in schizophrenia to reduce the impact of cognitive impairments on employment [37,43]. There is an extensive literature about the benefits of CR on work integration, but less is known about this relationship in the French context. Different types of vocational programs coexist in France: SE, which is an evidence-based practice to help people get competitive employment, social firms or ShE, and hybrid vocational programs [2]. Our study focuses on CR impacts in ShE. The expected benefits for participants are a better professional insertion and improvements in cognitive and social skills and functional outcomes.

## Methods

RemedRehab was a multicentric randomized comparative open trial in parallel groups conducted in eight centers in France between 2013 and 2018. This project is part of a strong partnership between the CR network and the ShE network in France. Participants were recruited into ShE firms before their insertion in employment (preparation phase).

A preselection of study candidates based on the following criteria was made prior to inclusion: scheduled entry in ShE 4 months later, age, native language, diagnosis, no neurological disorders (stroke, cranial trauma, and neurodegenerative diseases), no addiction (except tobacco), IQ > 70 (evaluated with Raven’s progressive matrices), and no treatment by electroconvulsive therapy in the previous 6 months. We included patients with schizophrenia based on DSM-IV criteria, aged from 18 to 45 years, speaking and reading French, whose clinical state was stable and who must integrate ShE positions within 4 months after inclusion. They were randomly assigned to one of two groups: cognitive training (RECOS group) and Treatment As Usual (TAU group). Randomization was stratified by site. Each intervention (RECOS and TAU) was conducted during 3 months before integration in ShE (preparation period).

Three evaluations were conducted by psychologists specializing in neuropsychology at T1, T2, and T3. Blinded outcome assessments were conducted. Participants benefited from CR or TAU during the preparation period (T1–T2) prior to their entry in ShE (T2). They were reevaluated at integration in ShE (T2), and a third evaluation was conducted 6 months later (T3). Evaluations were similar at T1, T2, and T3.

The aim of the study was to compare the benefits of the RECOS CR program versus the TAU on access to employment and work attendance for people with schizophrenia, measured by the following ratio (*R*): number of hours worked on number of hours stipulated in the contract of employment in the 6 months following the entry into ShE.

Secondary outcomes were:

1. Comparative evolution of cognitive outcomes in the RECOS and TAU groups at baseline (T1), at integration in ShE (T2), and 6 months later (T3). Assessments of cognitive functions were based on standardized neuropsychological tests intended to assess impaired cognitive functions in schizophrenia:
  1.1. Working memory: memory of figures (Wechsler Adult Intelligence Scale—Fourth Edition) [44,45];
  1.2. Spatial memory (MEM IV) [44];
  1.3. Selective attention and processing speed: D2 [46]; Stroop [47];
  1.4. Verbal memory: RL/RI-16 items [48]; RBMT [49];
  1.5. Verbal fluency: fluence [50];
  1.6. Memory and visual–spatial attention: BVMT [51];
  1.7. Reasoning: matrices [45];
2. Comparative evolution of the clinical and functional outcomes in the RECOS and TAU groups at baseline (T1), at integration in ShE (T2), and 6 months later (T3):
  2.1. Positive and Negative Syndrome Scale [52];
  2.2. Behavioral Activation for Depression Scale [53];
  2.3. Self-Appraisal of Illness Questionnaire;
  2.4. Self-Esteem Rating Scale [54];
  2.5. Warwick–Edinburgh Mental Well-Being Scale [55];
  2.6. French Social Autonomy Scale (EAS) [56];
  2.7. Ambiguous Intentions Hostility Questionnaire [57];
  2.8. MIC-CR Cognitive Insight Scale [58];
  2.9. Assessment of technical and interpersonal skills at work (EATR); (Annex 1).

In the RECOS group, patients benefited from specific CR by one training module (chosen with respect to the most impaired cognitive function, as measured in the neuropsychological assessment) among the five proposed by the program (reasoning, verbal memory, visual–spatial memory and attention, working memory, and selective attention). RECOS is a CR program designed for patients with a schizophrenia spectrum disorder [59,60]. It is acknowledged that cognitive deficits differ from one patient to another. Consequently, RECOS aims at providing individualized CR therapy. The training consists of the realization of paper-and-pencil exercises (allowing the development of adapted strategies) and computerized exercises of increasing difficulty. The therapeutic part of the program takes place at two sessions of 1 hour per week plus 1 hour of homework for 15 weeks (30 1-hour remediation sessions). Patients in the TAU group benefited from of the usual nonspecific preparation period prior to ShE inclusion for 3 months. Initial assessment and follow-up were identical in the RECOS and TAU groups.

Statistical analyses were conducted on the R Software (https://www.r-project.org) by Francq C. and colleagues. All the data from patients in their group of randomization were considered in these analyses, whatever the treatment they actually received (crossover) and whatever their future in the trial (withdrawal). We conducted an Intent-To-Treat analysis. Generalized estimation equations and multilevel analyses were conducted. This allowed to consider the impact of determined care sequences on the evolution of the quantitative parameters collected iteratively and, conversely, the way in which certain characteristics of people (cognitive and psychosocial) predict the nature of the evolution of their professional insertion.

## Results

Seventy-nine patients were included in the study between October 2018 and September 2019. Twenty-six participants withdrew of the study or were lost of sight before the collection of the main outcome in ShE. Fifty-three patients completed the study (30 RECOS + 23 TAU). Among them, 30 have a complete dataset.

The average age was 34.2 for RECOS and 36 for TAU, and there were 5 women and 25 men in the RECOS group and 4 women and 19 men in the TAU group. There were no significant difference between the two groups at baseline on other outcomes (Table 1).

**Table 1.** Characteristics of participants included with primary outcome evaluated (*n* = 53).

	*n*	Male	Female	Age (years)	Education level (years)	F-Nart
RECOS	30	25	5	34.2	10.8	102
TAU	23	19	4	36	10.8	102

*Abbreviation:* TAU, Treatment As Usual.

**Figure 1.**
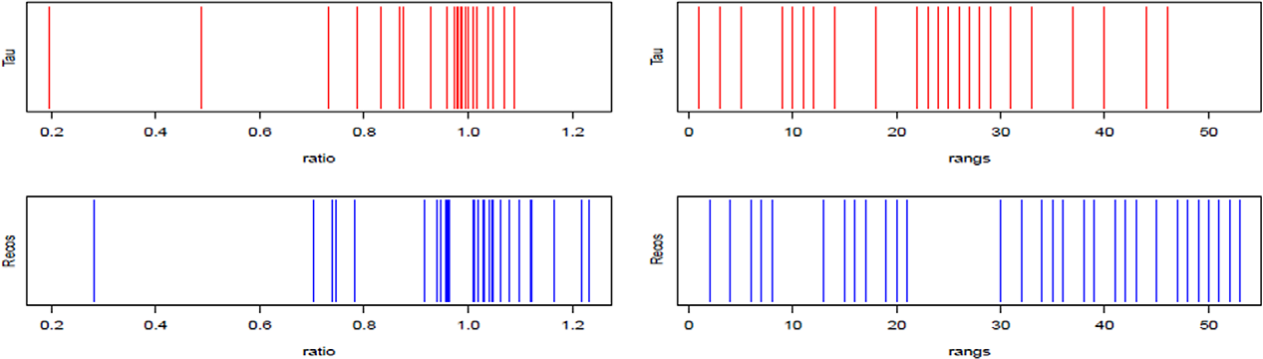
Distribution of the variable *R* (left) and its ranks (right) for the Treatment As Usual and RECOS groups.

Main outcome was collected for 53 of them. The values of *R* ≥ 1 were obtained from workers who held their contract (=) or worked overtime (>). *R* < 1 values were obtained from workers who showed unjustified absenteeism or who abandoned their job before the end of the 6-month follow-up. Better performances on the main outcome were observed for the RECOS group: values of the *R* ratio (hours worked / planned hours) equal to 1 or greater than 1 were significantly higher in the RECOS group than in the TAU group (Figure 1 and Table 2). The distribution of the ranks of these ratios confirms this observation in favor of the RECOS group (Figure 1).

**Table 2.** Difference in *R* average values (without outliers; *n* = 53).

m(TAU)	m(RECOS)	Difference	*p* value (Student test)	*p* value (Wilcoxon test)
0.938	1.004	0.066	0.0448	0.0328

*Abbreviation:* TAU, Treatment As Usual.

**Figure 2.**
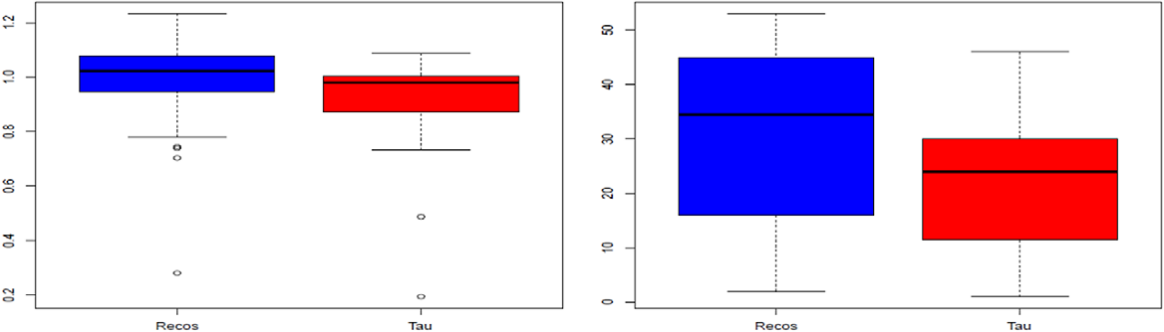
Boxplots of the variable *R* (left) and its ranks (right) for the Treatment As Usual and RECOS groups.

Boxplots of the distribution of *R* values (Figure 2) show a clear advantage for workers in the RECOS group, and this for the three quartiles [bottom of the box = first quartile or Q1; median = second quartile or Q2; top of the box = third quartile or Q3), or 75% of the total population. Extreme values (below the limit of the lower mustache) are more numerous in this arm (4 for RECOS against 2 for TAU). Among them, 2 (1 RECOS and 1 TAU) can be considered as “nonstandard.” The same observation (superiority of RECOS over TAU) can be made for the row values (Figure 2).

The difference in the ratios observed between the two groups is statistically significant with a risk of 10% for the total population analyzed (*n* = 53; Student and Wilcoxon–Mann–Whitney tests); it is also clearly significant at the risk of 5% if we eliminate the extreme values (1 in each group) corresponding to two patients with a poorly defined professional project (withdrawal from ShE after 2 or 3 months, with absenteeism therefore during these few weeks of follow-up; Table 2).

An analysis of variance was carried out to test the hypothesis that variables other than the main outcome had the same profile in the two groups at baseline (T1). For some of these variables and some individuals, we observed a lot of missing data. We therefore replaced these missing data with an empirical average calculated from the data available for other individuals. No significant difference was detected between the two groups at 5% risk, except for two variables (out of 57): the EAS (*p* = 0.04164) and the indirect span of the memory of figures (*p* = 0.01566). The population was therefore very homogeneous in the two groups at baseline (T1).

We then wanted to know if these variables had been influenced by the treatment provided. No difference in evolution between the two groups was observed for the majority of the variables considered, except for:

1. RBMT variables (verbal memory), PNEG (PANNS negative symptoms) and PPOS (PANNS positive symptoms), and EAS for which the scores are lower in the RECOS group compared to the TAU group, either between T1 and T2, T1 and T3, or both;
2. TMTA where the difference is significantly larger for RECOS than for TAU between T1 and T2.

We can also notice many “nonstandard” values for the variables *FR* (false recognition), *RC* (right recognition), and *Ind* (RC-FR) of the BVMT visual memory scale, which makes any analysis irrelevant.

## Conclusions

The objective of this study was to assess the impact of an individualized CR program on maintaining ShE for people with schizophrenia. The main outcome was work attendance, on the assumption that the presence of cognitive deficits would affect the motivation and efficiency of people in their workplace and therefore lead to more absenteeism. We have shown that participants benefited from a RECOS individualized CR program allows a better rate of work attendance (*R* ratio) in ShE, compared to the ones benefited from TAU. The population studied had just entered employment, as the 3-month intervention was delivered during the preparation period (around 4 months) before taking their position. Therefore, we tested the impact of an individualized CR program which was previously validated in this population [59,60] on professional insertion in the traditional or “train and place” vocational rehabilitation model still widely use in France [17–19].

The limitations of the study were the small number of participants compared to the number of subjects required calculated beforehand. In addition, 26 participants chose to withdraw of the study or were lost to follow-up before the first outcome was collected. In the end, the data of only 53 participants could be analyzed for the first outcome (30 RECOS + 23 TAU), and only 30 datasets were complete for the first and secondary outcomes. It was therefore difficult to conclude on secondary outcomes, and most results were not significant. The collection of secondary outcomes at baseline, however, allowed us to observe that the population was homogeneous on the different criteria between the two groups TAU and RECOS.

We can conclude from this study that traditional vocational rehabilitation or “train and place” paradigm enhanced with individualized CR in a population of patients with schizophrenia is efficient on work attendance during the first months of work integration. These observations are consistent with those observed in the literature. It is usually observed in the literature that people with a severe handicap get more opportunities in ShE than in competitive workplaces. People with schizophrenia working in competitive settings have lower symptoms, are less hospitalized, and have higher executive functions and higher functional capacity than people working in ShE or who are unemployed [24,61,62]. They are also more likely to find employment if they were already working before hospitalization. ShE has long been the only alternative for people with schizophrenia [19]. The effectiveness of SE in this population has now been demonstrated; however, ShE can still remain a useful alternative for people with schizophrenia and significant cognitive difficulties, for whom it is more difficult to access competitive work market. This hypothesis is to be moderated, because it has also been observed that at an equal level of symptoms, quality of life, and self-esteem, people who enter competitive employment have better improvement on these outcomes than people who enter ShE [12]. Above all, access to competitive employment for these people should be encouraged by making it more inclusive and allowing the necessary adaptations to persistent cognitive difficulties.

Further studies would be required to study the maintenance of the beneficial effects observed over time, both on cognitive parameters and on work attendance and other functional outcomes in ShE. Furthermore, RemedRehab studied the impact of the remediation of neurocognitive disorders in access and job maintaining in traditional vocational rehabilitation, but it would be interesting to study the impact of social cognition training on these same parameters.

## Data Availability

The datasets generated during and/or analyzed during the current study are available from the corresponding author on reasonable request.
